# Pharmacological and Expectancy Effects of a Low Amount of Alcohol Drinking on Outcome Valuation and Risk Perception in Males and Females

**DOI:** 10.1371/journal.pone.0154083

**Published:** 2016-04-21

**Authors:** Tomokazu Tsurugizawa, Shinsuke Tokuda, Tokiko Harada, Taiki Takahashi, Norihiro Sadato

**Affiliations:** 1 Institute for Innovation, Ajinomoto Co., Inc., Kawasaki, Japan; 2 NeuroSpin, Commissariat à l'Energie Atomique et aux Energies Alternatives, CEA Saclay, Gif-sur-Yvette, France; 3 Department of Behavioral Science, Graduate School of Letters, Hokkaido University, Sapporo, Japan; 4 Division of Cerebral Integration, National Institute for Physiological Sciences, Okazaki, Japan; Department of Physiological Sciences, SOKENDAI (The Graduate University for Advanced Studies), Okazaki, Japan; University of Chicago, UNITED STATES

## Abstract

The high-dose, alcohol-induced influences on risk perception and loss aversion depend on sex. On the other hand, low-dose alcohol has less effect on risky behavior. However, the effect of low-dose alcohol on subjective valuation of gain or loss and also the effect of placebo (expectancy of alcohol) on risk perception have not been fully investigated. We investigated the effects of low-dose alcohol (0.02 g/100 ml blood alcohol concentration) and placebo effects on subjective risk perception and subjective valuation of uncertain gain and loss in females and males. Participants in the control group and the placebo group were served alcohol-free, wine-flavored beverage and participants of alcohol group were served wine (14% alcohol). The placebo group was not informed that the drink was not alcohol but the control group was informed. Then paper–pencil tasks for subjective risk perception and valuation of gain or loss were performed 45 min after drinking the beverage. The participants were asked to draw the line on a 180 mm scale for each question. The placebo effects as well as the low-dose alcohol effects were observed in subjective valuations of gain or loss. Except for effect of beverages, a gender difference was also observed for subjective likelihood. The females estimated a low-probability loss as more likely and estimated a high-probability gain as less likely than did the males. From the Stevens’ law fitting analysis, the placebo, not alcohol, significantly induced the psychophysical effect of the subjective valuation of gain or loss. These results indicate that the psychological effects of expectancy of alcohol (placebo) could be a major factor in changing the subjective valuation of gain or loss over the pharmacological effects of a small amount of alcohol (like a glass of wine). Furthermore, these results also indicate that gender differences should be taken into account when investigating pharmacological or psychological effect on decision-making.

## Introduction

There is social concern regarding not only increased risky behavior but also increased health risks from alcohol consumption, especially alcohol abuse. Alcohol is positively correlated with making risky choices and impulsive decisions, resulting in lost gambles, traffic accidents, or violence. Alcohol dose-dependently affects brain function, resulting in more risky choices [1], and low dose of alcohol drinks (i.e., a glass of wine) do not seem to affect several variables such as cognitive load [2], cognitive control [3] and visuomotor function [4]. However, given that we often have to act under the influence of low dose of alcohol, e.g., at a party, it should be understood how low dose of alcohol affect risky decisions. In addition to the pharmacological effect of alcohol, the psychological expectancy effect of alcohol, which is called a “placebo” effect, should be investigated in regard to risky behavior [5].

Recently, it has become possible to evaluate varying degrees of risky choices in the laboratory environment in a lot of social behavioral studies. In the developing field of neuroeconomics, several parameters related to risky decisions have been associated with neuropsychological variables [6]. In neuroeconomics study, the risky decisions are characterized by two behavioral and psychological parameters: underestimating the risk perception of negative consequences and sensitivity to punishment (often in addition to overestimating the likelihood of positive consequences and/or high sensitivity to reward) [7]. Previous studies have also demonstrated a gain-loss asymmetry in risk perception by utilizing the psychophysical measurement of risk perception and the subjective valuation of gain (reward) and loss (punishment) [8, 9]. The subjective theory of value is a theory of value that advances the idea that the value is not determined by any inherent property but instead the value is determined by individual places on the achievement of his desired ends [10]. Loss aversion, the idea that negative valuations have a higher psychological impact than positive valuations, is considered an important variable in consumer research [12] and behavioral economics [13], in addition to neuroeconomics [14]. Loss aversion leads to risk aversion when people evaluate outcomes comprising similar gains and losses, because people prefer avoiding losses over making gains. Interestingly, the subjective valuation of loss aversion is different between females and males: females are more aversive to loss aversion rather than males [11]. Previous studies have focused on risk perception in alcohol consumers. As expected, risk perception is negatively proportional to alcohol consumption [15], but there is no study that has investigated the effect of alcohol consumption on loss aversion and gain happiness, i.e., hypersensitivity to negative outcomes and hyposensitivity to positive outcomes.

In the present study, we quantitatively investigated the effect of alcohol and placebo on risk perception and subjective valuation of gains and losses in young, healthy participants by utilizing behavioral and psychophysical paradigms established in our previous studies. This approach is important for establishing more efficient behavioral and medical treatments for alcohol abuse and related health problems, because recent studies have proven the advantages of treatments based on behavioral economics [16–18].

## Materials and Methods

### Participants

Seventy-five right-handed, nonsmoking and healthy university students participated in this study. Participants were randomly assigned to alcohol, placebo, or control group (Table 1). There were no significant differences in the age and body weight of the participants. They consumed alcohol at a frequency of less than once per week in all groups. All subjects voluntarily signed informed consent statements, in accordance with the ethics committee of the National Institute for Physiological Sciences, which approved this study.

**Table 1:** Average age and body weights of the participants in the alcohol, placebo, and control groups.

Group	N	Age	Body weight (kg)	Alcohol consumption days / week
Alcohol—Male	14	23.4 ± 2.1	60.7 ± 7.6	0.6 ± 0.1
Alcohol—Female	9	22.3 ± 4.4	50.0 ± 4.5	0.6 ± 0.2
Placebo—Male	15	22.5 ± 4.0	62.0 ± 7.1	0.8 ± 0.2
Placebo—Female	8	23.1 ± 6.0	52.5 ± 6.3	0.4 ± 0.2
Control—Male	18	23.6 ± 5.3	66.5 ± 10	0.7 ± 0.2
Control—Female	11	22.6 ± 2.6	51.0 ± 8.0	0.2 ± 0.4

Data are expressed as the mean ± SEM.

### Beverages

Participants in the control group and the placebo group were served an alcohol-free, wine-flavored grape juice, while participants in the alcohol group were served a French red wine (14% alcohol). Each participant received an individually tailored dose of their alcoholic or nonalcoholic beverage; individual dose was calculated based on weight and sex, following a Widmark formula [19] to reach estimated blood alcohol concentration (BAC) targets of 0.02 g / 100 ml (89.3 ± 3.7 ml, averaged for all participants). Subjects in the placebo group and alcohol group were not informed that the beverage was a nonalcoholic drink or an alcoholic drink during the experiment; instead, an opened, labeled, real wine bottle containing wine was placed on the table. Thus, they took a nonalcoholic or alcoholic drink while seeing this bottle of real wine. It was also confirmed that the volunteers in the placebo and alcohol groups recognized a nonalcoholic beverage as a real wine after drinking. The subjects in the control group were informed that it was nonalcoholic drink before drinking. The breath alcohol levels after 45 min of alcohol drinking were measured in the preliminary study to confirm the alcohol levels and it was not measured in the volunteers for the reason as described in Discussion.

### Procedure

**Fig 1:**
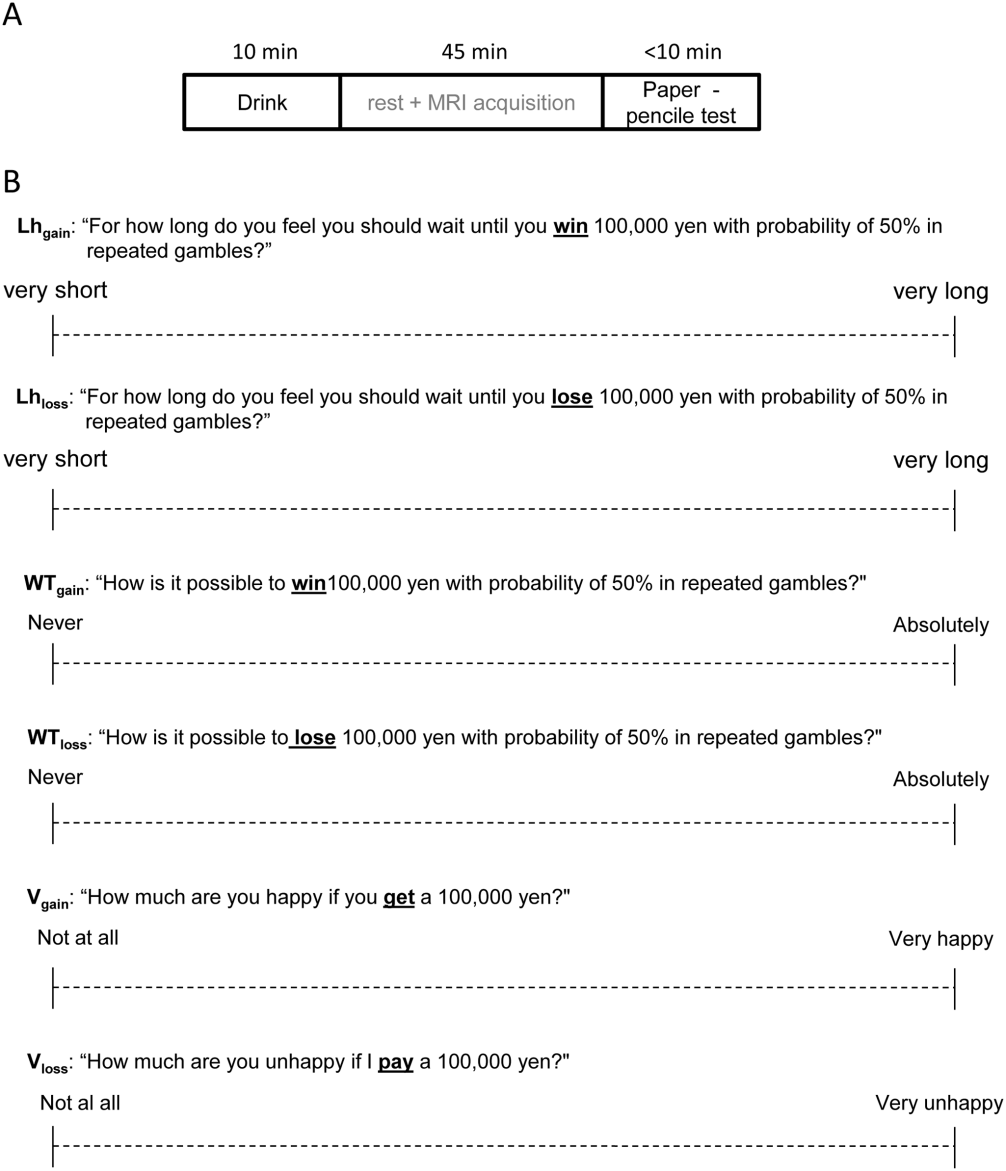
Experimental paradigm and Example of paper–pencil task. (A) Diagram of the experimental paradigm. (B) The participants were asked to draw a line on a 180-mm scale, from the left anchor to the right, to indicate the length of subjective likelihood, subjective waiting time and subjective valuation in response to each question.

The subjects were asked not to eat one hour before the start of the experiment to avoid the effect of food on alcohol absorptivity. The subjects drank the beverage before the MRI scanning sessions (around 45 min) over a short time (10 min), and then the paper-pencil tasks (within 10min) were performed after drinking the beverage (Fig 1A). In the present study, we analyzed the paper-pencil tasks independent of MRI experiments.

### Risk perception tasks

To examine participants’ risk perception, we assessed the subjective perception of the likelihood of uncertain gain (Lh_gain_) and loss (Lh_loss_) (± 100,000 yen (hypothetical), approximately equal to $1,000, on a 180 mm scale) at seven probabilities of 0.05, 0.1, 0.3, 0.5, 0.7, 0.9, and 0.95. Additionally, to further assess risk perception by utilizing the nonlinear time perception theory of decision under risk [20], we asked participants for their subjective perception of the waiting time for both uncertain gain (WT_gain_) and loss (WT_loss_) with seven probabilities (0.05, 0.1, 0.3, 0.5, 0.7, 0.9, and 0.95), as described in previous studies [8, 21] (e.g., “For how long do you feel you should wait until you win 100,000 yen with probability of 50% in repeated gambles?” for the gain task; “For how long do you feel you should wait until you lose 100,000 yen with probability of 50% in repeated gambles?” for the loss task). Participants were asked to draw a line on a 180 mm scale to indicate the subjective waiting time or subjective likelihood (minimum of 0 mm and maximum of 180 mm) until receiving reward (Fig 1B).

### Subjective valuation of gain happiness and loss aversion

The subjective valuation of the gain happiness (V_gain_) and loss aversion (V_loss_) was assessed in participants by drawing a line on a 180 mm scale in the same manner as risk perception, to their gain happiness and loss aversion in the case of 10 different amounts of hypothetical money (10,000, 20,000, 30,000, 40,000, 50,000, 60,000, 70,000, 80,000, 90,000, and 100,000 yen) (Fig 1B).

### Psychophysical models of risk perception

The differences in risk perception and subjective valuation were assessed using psychophysical models (Stevens’ power model) to examine the psychological effects of nonlinearities in risk perception [22]. The general form of the law is written as *Ψ*(*x*) = *ax*^*s*^, where *Ψ*(*x*) is the subjective magnitude of the sensation evoked by the stimulus, *x* is the magnitude of the physical stimulus, s is an exponent that depends on the type of stimulation, and *a* is a proportionality constant. The concavity of *Ψ*(*x*) can be expressed as follows:

$$concavity\;of\;\Psi \left(x\right)\;:=\;-\frac{\frac{d^{2}}{dx^{2}}\Psi \left(x\right)}{\frac{d}{dx}\Psi \left(x\right)}=\left(1-s\right)x^{-1}$$ (1)

Given that *x* would be constant among three groups, (1-s) is proportional to *Ψ*(*x*). If the s-value is close to 1, *Ψ*(*x*) is approximately linear and thus there is no nonlinearly distorting psychological effect. If s < 1, (1) the psychological likelihood or waiting time has diminishing sensitivity to *x*; i.e., *Ψ*(*x*) is an increasing function of *x* (because *Ψ′*(*x*) > 0), but an increase in *Ψ*(*x*) in response to an increase in x (*Ψ′′*(*x*)< 0) is a decreasing function of *x* (this property is referred to as “marginally decreasing” when *x* is an amount of reward) and (2) nonlinearity in gain happiness/loss aversion increases as the s-value decreases. It is to be noted that in standard economic theory, the concavity of *Ψ*(*x*) as defined in eq (1) corresponds to risk attitude if the agent is rational [23]. In contrast, if the agent is not fully rational (“bounded rational”), the concavity of subjective perception of risk (in terms of likelihood and/or waiting time) also influences the agent’s risk attitude [20]. The advantage of our present analytical strategy is that we can fully capture the characteristics of participants’ decisions under risk by utilizing the psychophysical models. The goodness-of-fit of Stevens’ nonlinear power model was compared to a linear model (s = 1) using the Akaike information criterion (AIC) as described in Takahashi et al. [21]. The AIC is the most standard criterion for the fitness of mathematical models for observed data when the sample size is small. Briefly, the AIC can be calculated as follows:

AIC=N⋅ln[(residual sum of squares)/N]+2k

where N is the sample size and k is the number of parameters to be estimated.

### Statistics

The three-way (2 x 3 x 2) analyses of variance (ANOVAs) followed by post-hoc Holm multiple comparisons were performed with the sex (males and females; between-subject factor), beverage (alcohol, placebo, and control; between-subject factor), and income signs (gain and loss; within-subject factor) as the between-subject variables to reveal significant main-factor effects or interactions.

## Results

### Comparison between Stevens’ power law and a linear model

Although Stevens’ nonlinear power law model has already been shown to fit well to risk perception and subjective valuations [8, 21], it would be worthwhile to compare it with a linear model (Table 2). The AICs of Stevens’ power law model were significantly lower than those of the linear model in all tasks (Lh, WT, and V), indicating that this model had a better suitability compared to linear model in all tasks.

**Table 2:** AICs by linear and non-linear Stevens’ power law.

	Model	AIC
Lh_gain_	Linear	52.80 ± 0.90
	Nonlinear	*50.42 ± 0.91
Lh_loss_	Linear	79.37 ± 0.64
	Nonlinear	*64.46 ± 0.72
WT_gain_	Linear	62.89 ± 1.00
	Nonlinear	*54.92 ± 0.74
WT_loss_	Linear	75.64 ± 0.88
	Nonlinear	*60.07 ± 0.95
V_gain_	Linear	114.02 ± 0.78
	Nonlinear	*68.66 ± 1.85
V_loss_	Linear	110.03 ± 1.21
	Nonlinear	*72.82 ± 1.97

Data are expressed as the mean ± SEM. The AIC was calculated from all participants. The AICs of linear vs. nonlinear model were compared within each parameter.

* *P* < 0.01 by Student’s *t*-test.

### Subjective perception of likelihood

**Fig 2:**
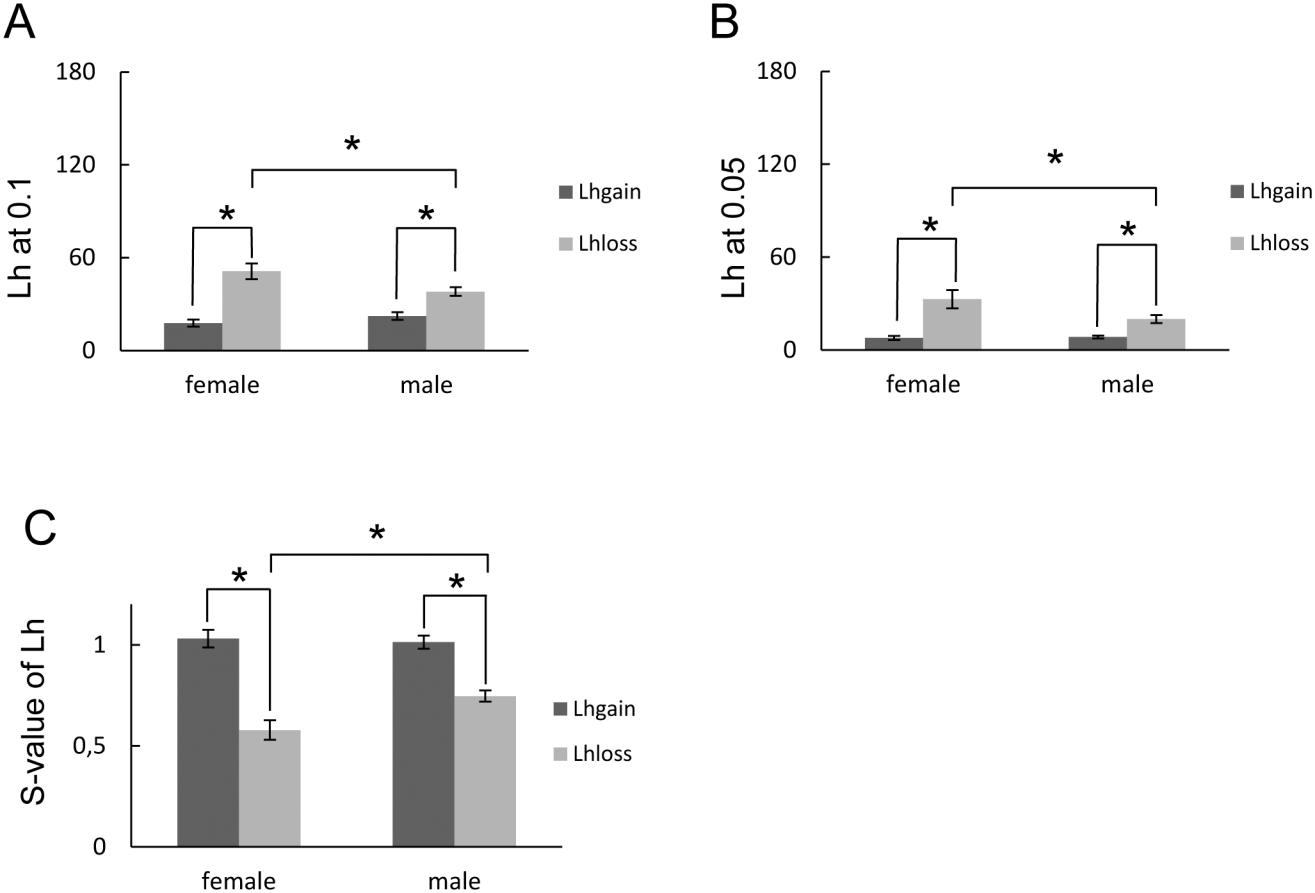
Subjective likelihood of gain and loss. Lh at a probability of (A) 0.1 and (B) 0.05. (C) s-vales of the Lh_gain_ and Lh_loss_ in each sex. * *P* < 0.05, post-hoc Holm multiple comparisons following ANOVA. Data are expressed as mean ± SEM. Vertical axis: a 180-mm scale of the paper–pencil task.

The statistical significance of subjective likelihood is shown in Table 3. There was significant interaction between sex and sign at probabilities of 0.10 (F(1, 59.64) = 8.86, *P* = .004) and 0.05 (F(1, 45.21) = 4.91, *P* = .032). At these probabilities, the Lh_loss_ was significantly higher than the Lh_gain_ both in females and in males (Fig 2A and 2B). Furthermore, the Lh_loss_ in females was also significantly higher than that in males at these probabilities (Fig 2A and 2B). At higher probabilities (0.95 and 0.90), the Lh in males was higher than that in females (Table 3). The main factor effect in the sign was also observed at 4 probabilities between 0.90 and 0.30 (Table 3). At these probabilities, the Lh_gain_ was lower than the Lh_loss_. The s-values were also calculated from the Lh_gain_ and the Lh_loss_ (Table 3). There was significant interaction between sex and income signs (gain/loss) (F (2, 63) = 7.57, *P* = 0.0076). The s-values of the Lh_loss_ in males were significantly higher than those in females (Fig 2C). The s-values of Lh_gain_ were significantly higher than the Lh_loss_ both in females and in males (Fig 2C).

**Table 3:** Statistical significances in subjective likelihood.

	Subjective likelihood			
	Factors			Interaction
	Sex	Drink	Sign	
0.95	* F < M	-	-	-
0.90	* F < M	-	* G < L	-
0.70	-	-	* G < L	-
0.50	-	-	* G < L	-
0.30	-	-	* G < L	-
0.10	-	-	***	Sex x Sign
0.05	*	-	*	Sex x Sign
s	*	-	*	Sex x Sign

* *P* < 0.05,

*** *P* < 0.001 by post-hoc Holm multiple comparisons following ANOVA.

F: females; M: males; G: gain; L: loss.

### Subjective perception of waiting time

The statistical significance of subjective waiting time is shown in Table 4. There was no significant interaction among the sex, drink, and income signs for WT. The WT_gain_ was significantly longer than the WT_loss_ at probabilities between 0.05 and 0.70. The s-values were also calculated from the WT_gain_ and WT_loss_ (Table 4). There was no significant difference by ANOVA.

**Table 4:** Statistical significances in subjective waiting time.

	Subjective waiting time			
	Factors			Interaction
	Sex	Drink	Sign	
0.95	-	-	-	-
0.90	-	-	-	-
0.70	-	-	** L < G	-
0.50	-	-	** L < G	-
0.30	-	-	*** L < G	-
0.10	-	-	*** L < G	-
0.05	-	-	*** L < G	-
s	-	-	-	-

** *P* < 0.01,

*** *P* < 0.001 by post-hoc Holm multiple comparisons following ANOVA.

G: gain; L: loss.

### Subjective valuation of gain happiness and loss aversion

**Fig 3:**
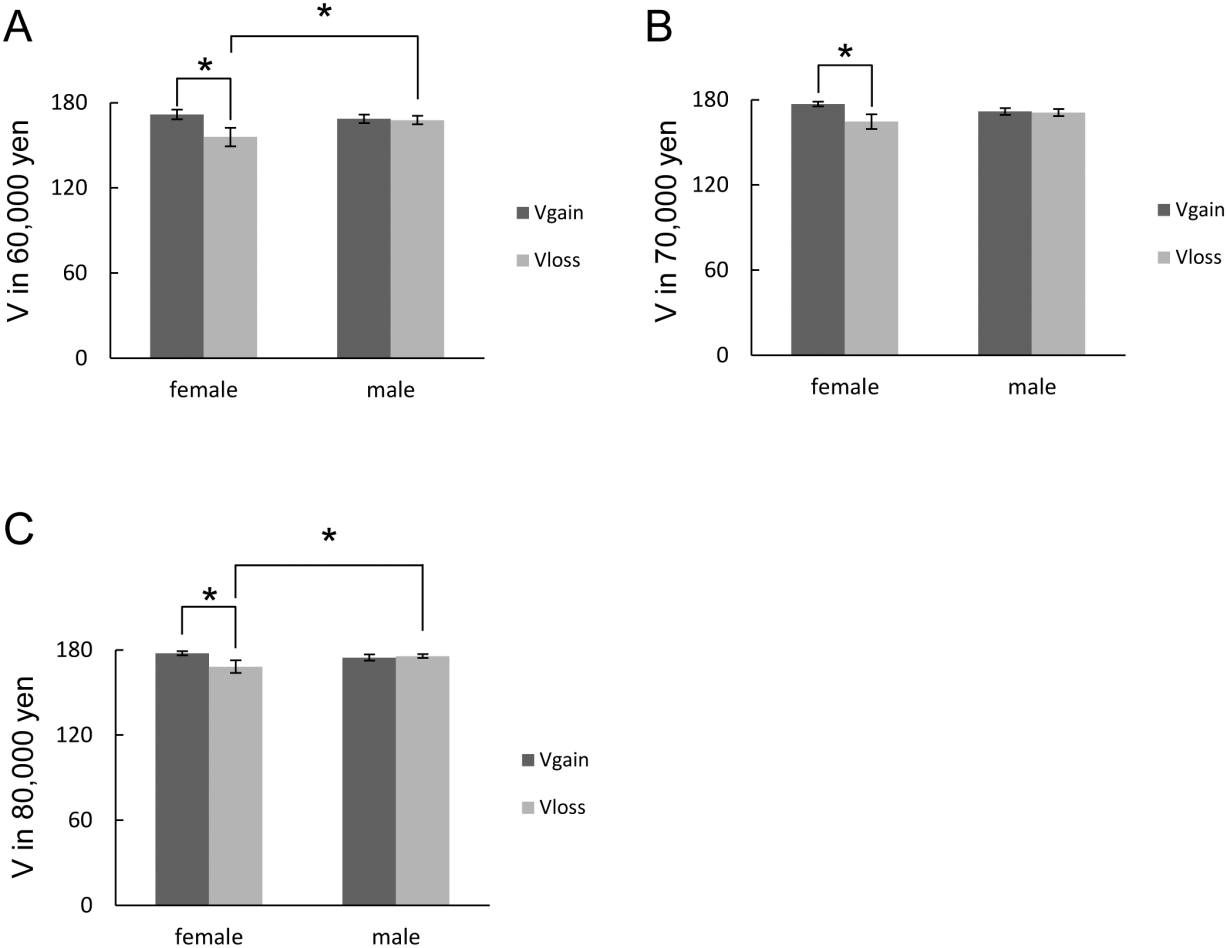
Subjective valuation of gain happiness and loss aversion. V of (A) 60,000 yen, (B) 70,000 yen and (C) 80,000 yen. * *P* < 0.05, post-hoc Holm multiple comparisons following ANOVA. Data are expressed as mean ± SEM. Vertical axis: a 180 mm scale of the paper–pencil task.

The statistical significance of the subjective likelihood is shown in Table 5. In contrast to subjective likelihood and waiting time, there were significant main-factor effects for the income sign and beverages. The subjective values in the alcohol group with a low-to-moderate amount (between 10,000 and 50,000 yen) were lower than those in the control group. The subjective values in the placebo group with a low amount (10,000 and 20,000 yen) were also lower than those in the control group. Furthermore, the V_loss_ with a low amount (10,000, 20,000, and 30,000 yen) was lower than the V_gain_. A significant interaction was observed with moderate amounts (between 60,000 and 80,000 yen) (F(1,58.25) = 4.79, *P* = .033 for 60,000 yen; F(1,53.17) = 4.90, *P* = .031 for 70,000 yen; and F(1,47.91) = 5.58, *P* = 0.022 for 80,000 yen). The V_gain_ in females was higher than the V_loss_ in females for amounts between 60,000 and 80,000 yen (Fig 3A–3C). The V_loss_ for 60,000 or 80,000 yen in males was higher than that in females. The s-values of the V_gain_ and the V_loss_ were also analyzed. Although there was no significant interaction among the factors, there were significant main-factor effects for the income sign and beverages (Table 5). The s-value for the V_loss_ was significantly higher than that for the V_gain_. The s-value in the placebo group was also higher than that in the control group.

**Table 5:** Statistical significances in subjective value.

	Subjective value			
	Factors			Interaction
	Sex	Drink	Sign	
10,000 yen	-	** A < C, P < C	** L < G	-
20,000 yen	-	** A < C, P < C	** L < G	-
30,000 yen	-	* A < C	* L < G	-
40,000 yen	-	* A < C	-	-
50,000 yen	-	* A < C	-	-
60,000 yen	-	-	*	Sex x Sign
70,000 yen	-	-	*	Sex x Sign
80,000 yen	-	-	-	Sex x Sign
90,000 yen	-	-	-	-
100,000 yen	-	-	-	-
S	-	* P > C	** L < G	-

* *P* < 0.05,

** *P* < 0.01 by post-hoc Holm multiple comparisons following ANOVA.

F: females; M: males; A: alcohol; O: placebo; C: control; G: gain; L: loss.

## Discussion

The current study clearly showed not only beverage effects (alcohol, placebo) but also gender differences in subjective risk perception and subjective valuation of gain or loss. Only the placebo induced the nonlinear psychophysical effects of the subjective valuation of gain or loss, but this effect was not sex dependent. Our data also indicate that concurrent comparisons of behavioral and psychophysical functions between females and males are required for the comprehensive understanding of risky decisions, given that there is gender-dependent difference in subjective valuation.

### General gender difference

Gender differences were observed in subjective likelihood and subjective valuation. The present study showed that females estimated a low probability of loss as more likely and estimated a high probability of gain as less likely rather than males did (Table 3 and Fig 2). This indicates that females consider uncertain gains and losses as more risky than males. These results are consistent with previous studies showing that females take fewer risks like gambling and estimate a higher probability of negative consequences [24–26]. However, the current results of subjective valuation also showed that females were less averse to moderate amounts of loss compared to males (Fig 3). It does not match the evidence that females are more loss-averse in investment [11]. However, it is possible that the subjective risk perception of “hypothetical” money could link to actual behavioral results more closely than the subjective valuation of loss aversion of hypothetical money. Further research should be performed to elucidate this discrepancy with a task using actual money gains or losses. Remarkably, the present study first investigated the gender difference of the subjective valuation task of gain or loss. Although there still remained the ambiguity to compare gain happiness and loss aversion, as discussed later, this asymmetry of the subjective valuation of gain or loss due to gender will be valuable in considering gender differences in risky decisions.

### Alcohol and placebo effects on subjective valuation

Previous reports have shown that the consumption of high dose of alcohol (BAC > 0.06 g / 100 ml) induces an increase in loss aversion in females [27]. Additionally, risk perception is negatively related to alcohol consumption [15]. These reports correspond to the results in the current study. In the current study, given that subjects consumed low dose of alcohol, the expected pharmacological effect of alcohol was relatively smaller compared to those from heavy alcohol consumption studies. Instead, there might be mainly a psychological expectancy effect, where the subject recognizes the drinks as alcohol (placebo effect) [5, 28]. Although this effect is often weak and vulnerable, they are observed often appear to be due to the individual attempting to compensate for the expected effects of the alcohol [5]. A functional magnetic resonance study also reveals that the brain activation of subjects with low amounts of alcohol (0.05% breath alcohol concentration) is similar to that of the placebo group in cognition tasks but not to control [3], but there has been no evidence of functional MRI regarding risky choices. The current results suggest that functional imaging of placebo and low-dose alcohol effects should be performed with subjective valuation tasks, as these are more sensitive to psychological effects.

In the present study, we performed 3-way ANOVAs to compare the effect of sex, beverages, and income signs at each probability. However, because it was thought to be helpful to compare the statistical analysis with a 4-way repeated-measures ANOVA (sex, beverages, income, and probabilities), we additionally analyzed our data (S1 Table). Consequently, essentially the same conclusions as the 3-way ANOVA were obtained.

### Beverage effects and gender factors in the linearity of psychophysical function

The previous study shows good-fitness of Stevens’ power model, which indicates the nonlinear distortion of psychological times and happiness gain for the risks [21, 29, 30]. In the present study, we first successfully applied this model to the pharmacological effect and psychological effect of alcohol on a subjective valuation task, as well as risk perception. Interestingly, the s-value of the subjective valuation was significantly affected in the placebo group, but not the alcohol group, compared with the control group and there was no significant difference between males and females. This indicates that the placebo effect induced the anomalies of subjective valuation more significantly than low-dose alcohol. The placebo effect observed in this study could be also linked to the changes in mood or emotion in the group with a small amount of alcohol.

Although the beverage effect was not observed for risk perception, sex difference and asymmetry of income sign were observed for subjective likelihood. The significant distortion of the s-value of the Lh_loss_ compared with that of the Lh_gain_ both in females and males (Fig 2C) would indicate asymmetry of risk perception between gain and loss for the risk. Furthermore, this nonlinear distortion of the Lh_loss_ was more significant in females than males. Remarkably, in contrast with likelihood, there was no sex difference in waiting time, indicating that time perception of uncertain risk was independent of sex.

### Limitations and future directions

The limitations of the experiment in the present study are as follows. First, the alcohol levels of several subjects had been confirmed in the preliminary study using breath measurement apparatus. Then, to avoid volunteers in the placebo group knowing their drink was nonalcoholic when they saw the result of the measurement, we did not measure breath alcohol levels in the experiment. However, because of the limitation of the alcohol detection capability of the breath measurement protocol and lack of measurement in the volunteers, it is not completely clear that their blood alcohol levels were correctly 0.02%. Second, the same scale (180 mm) was used to assess V_gain_ and V_loss_ in this study. However, the equivalency between V_gain_ and V_loss_ is not clear. It should be investigated in future study. Third, the university students were selected as volunteers, but there might be a small difference of socioeconomic status among the students. Although it seems to be inconsequential, it should be mentioned in this section.

## Conclusion

The current study demonstrates that expectation of alcohol consumption influences risky behavior. Compared to previous reports with high amounts of alcohol, the pharmacological alcohol effects are small; instead, the psychological placebo effects were more significant. We should take this placebo-induced modification of risky choice into account because, in some cases, low-dose alcohol consumption is required in social activity, i.e., during parties and gambling.

## Supporting Information

S1 Table Statistical significances in Lh, WT, V. (DOCX)

## Abbreviations

AIC: the Akaike information criterion with small sample correction
BAC: blood alcohol concentration
Lh: likelihood
V: valuation
WT: waiting time
