# Supplementary material for: Pharmacological and Expectancy Effects of a Low Amount of Alcohol Drinking on Outcome Valuation and Risk Perception in Males and Females
Source: PLoS One. 2016 Apr 21;11(4):e0154083. doi: 10.1371/journal.pone.0154083 (PMC4839653; doi:10.1371/journal.pone.0154083)
Supplement: S1 Table — (DOCX) [file pone.0154083.s001.docx]

**Supporting Table 1: Statistical significances in Lh, WT, V.**

|  | Subjective waiting time | | | | |
| --- | --- | --- | --- | --- | --- |
|  | Factors | | | | Interaction |
|  | Sex | Beverage | Sign | Prob |  |
| Lh | *F_P1_ < M_P1_  *F_P2_ < M _P2_  *F_P7_ > M_P7_ | - | ***G<L _P1_  *** G<L _P2_  ** G<L _P3_  ** G<L _P6_  *** G<L _P7_ | For every probability P,  if Pi > Pj，then  Lh_Pi_ > Lh_Pj_ | Sex x Prob  Sign x Prob |
| WT | - | - | *** G>L_Female_  ** G>L_Male_  ***G>L _P1_  *** G>L _P2_  **G>L _P6_  ** G>L _P7_ | For every probability P,  if Pi > Pj，then  WT_Pi_ < WT_Pj_ | Sex x Sign  Sign x Prob |
| V | - | *A_A1_ < C_A1_  *P_A1_ < C_A1_  *A_A2_ < C_A2_  *P_A2_ < C_A2_  *A_A3_ < C_A3_  *A_A4_ < C_A4_  *A_A5_ < C_A5_ | ** G>L _A1_  ** G>L _A2_  * G>L _A3_  * G>L _A6_  ** G>L _A7_ | For every amount Yi,  if Yi > Yj，then  V_Yi_ ≧ V_Yj_ | Beverage x Amont  Sign x Amount |

*p < 0.05, **p < 0.01, ***p<0.001 by simple effect test following ANOVA. F, females; M, males; G, gain; L, loss; P1, probabilities 0.95; P2, probabilities 0.90; P3, probabilities 0.70; P4, probabilities 0.50; P5, probabilities 0.30; P6, probabilities 0.10; P7, probabilities 0.05; A1, amount 10000 yen; A2, amount 20000 yen; A3, amount 30000 yen; A4, amount 40000 yen; A5 amount 50000 yen; A6, amount 60000 yen; A7, amount 70000 yen; A8, amount 80000 yen; A9 amount 90000 yen; A10, amount 100000 yen.

e.g. A_A1_, Alcohol at 10,000 yen; P_A1_, Placebo at 10,000 yen; C_A1_, Control at 10,000 yen.
